# Deuterated carbohydrate probes as ‘label-free’ substrates for probing nutrient uptake in mycobacteria by nuclear reaction analysis†
†Electronic supplementary information (ESI) available. See DOI: 10.1039/c4cc09588j



**DOI:** 10.1039/c4cc09588j

**Published:** 2015-02-19

**Authors:** R. Lowery, M. I. Gibson, R. L. Thompson, E. Fullam

**Affiliations:** a School of Life Sciences , University of Warwick , CV4 7AL , UK . Email: e.fullam@warwick.ac.uk; b Department of Chemistry , University of Warwick , Gibbet Hill Road , Coventry , CV4 7AL , UK; c Department of Chemistry , Durham University , South Road , DH1 3LE , UK

## Abstract

Understanding and probing small molecule uptake in cells is challenging, requiring sterically large chemical labels, or radioactive isotopes. Here, the uptake of deuterated sugars by *Mycobacterium smegmatis*, a non-pathogenic model of *Mycobacterium tuberculosis*, has been investigated using ion-beam (nuclear reaction) analysis demonstrating a new technique for label-free nutrient acquisition measurement.

## Abstract 2

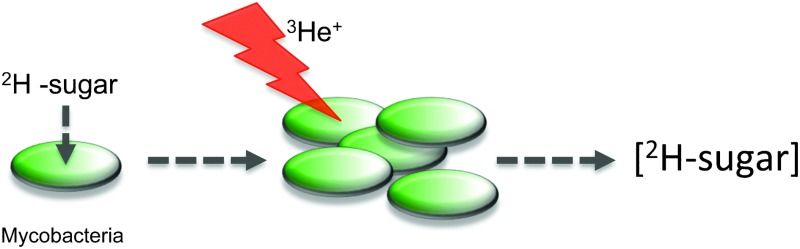
Deuterated sugars that are transported into mycobacteria can be detected using ion beam (nuclear reaction) analysis.

*Mycobacterium tuberculosis* is the etiological agent of tuberculosis (TB). TB remains a leading cause of death worldwide and in 2013 there were 9.0 million new cases and 1.5 million people died from TB.^1^ *M. tuberculosis* has a complex, unique cell wall that is rich in diverse carbohydrates and lipids that protects the bacterium from environmental stresses and chemotherapeutic agents. Despite the global threat of TB there are limited studies to investigate the nutrient requirements of this organism. Recent studies have implicated a role for putative sugar-transporters in *M. tuberculosis* to have an essential role during intracellular infection.^2^ Despite the obvious importance in gaining a detailed understanding of how *M. tuberculosis* processes carbohydrates, there exist very few detailed studies. This is due to the inherent lack of chromophore/fluorophore moieties on the sugars that significantly limits the analytic tools available to probe these essential biological processes *in vitro* and *in vivo*. Probes for such studies to date have been limited to radiolabelled,^2^ fluorescently modified,^3^ or azido-modified carbohydrates.^4^ Radiolabelled ^14^C/^3^H carbohydrates are expensive and non-standard carbohydrates are not readily available from commercial sources nor easy to synthesise. Fluorescently labelled carbohydrates have been used for such studies.^3^ However, their synthesis is often non-trivial and, more importantly, the large size of the fluorophores gives significant changes to the molecule. Extrapolating the function of the native carbohydrate from such derivatives is challenging, and non-specific uptake due to the lipophilic character of most dyes cannot be ruled out. The use of azido-sugars to metabolically label cells followed by Cu-free click,^5^ or Staudinger-ligation^6^ chemistry has been successfully undertaken.^7^ However, this method requires chemical synthesis of the desired azido-sugar, and pre-requisite knowledge about the intracellular processing of the sugar to ensure the structural modification will not influence its metabolism, relative to the native carbohydrate.^8^

Considering the above challenges, alternative biochemically passive labelling strategies are required. Deuterium is a stable, safe and readily available isotope of hydrogen that has near identical chemical reactivity, and has a low natural abundance in water of <0.02%, essential for any analytical method. We therefore reasoned that ^3^He Nuclear Reaction Analysis (NRA), which is uniquely sensitive towards the detection of deuterium could be employed to monitor the uptake of deuterated nutrients into bacteria.^9^ NRA is one of a family of MeV ion beam analysis techniques often used for quantitative materials analysis and depth profiling.^10,11^ The ^2^H(^3^He,p)α nuclear reaction is well established in materials analysis to quantify variation in ^2^H content with depth.^12,13^ This method is also appealing as data acquisition is rapid (typically less than 10 minutes) and does not rely on external calibration.

Here we have employed nuclear reaction analysis to study the uptake of deuterated carbohydrates by the non-pathogenic model organism *Mycobacterium smegmatis* (Fig. 1).

**Fig. 1:**
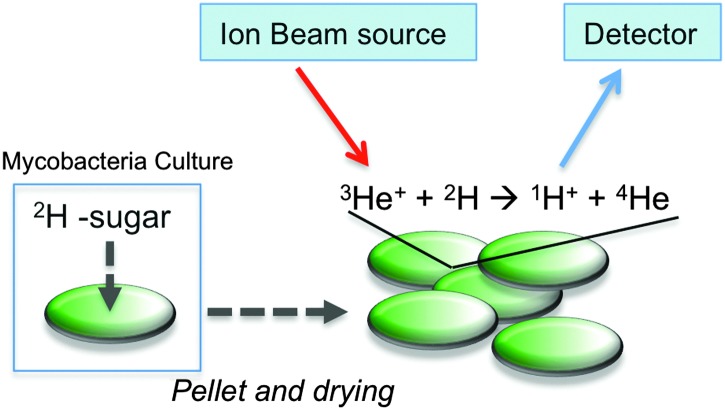
Schematic illustration for assessing ^2^H-carbohydrate uptake by *M. smegmatis* and ion beam analysis.

To obtain deuterated carbohydrates, the regio- and stereo-selective method of Sawama *et al.* was employed (Scheme 1).^14^ Briefly, the carbohydrates were dissolved in D_2_O, which serves as both solvent and the source of deuterium ions. Ruthenium on carbon (10 mol%) was added as the catalyst, and the reaction heated under a H_2_ atmosphere. In the case of the reducing sugars: glucose; galactose; mannose and arabinose it was necessary to use their anomeric methyl ethers, since previous studies have shown that non-methylated protected sugars undergo decomposition under these reaction conditions with Ru/C-catalysed hydrogenation possibly due to the hemi-acetal moiety of non-reduced sugars. For the non-reducing sugar trehalose this was not necessary. Following isolation, the modified carbohydrates were analysed by ESI-mass spectrometry and ^1^H NMR to estimate the degree of deuteration, summarised in Table 1 and also in the ESI.†

**Scheme 1:**
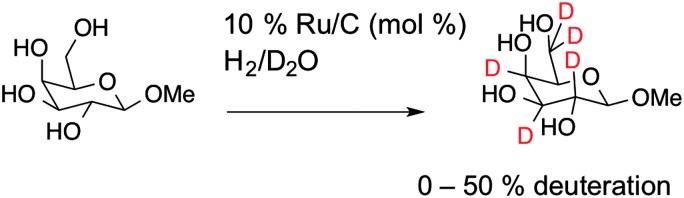
Synthetic scheme for carbohydrate deuteration, shown for 1-*O*-β-methyl galactose.

**Table 1:** Deuterated carbohydrates

Carbohydrate	*M*_R_ (^1^H)^*a*^	*M*_R_ (^2^H)^*b*^	% ^2^H^*c*^ (NMR)
Glu-OMe	194	217–220	67
Gal-OMe	194	217–219	40
Man-OMe	194	217–219	51
Arab-OMe	164	188–190	49
Trehalose	342	365–369	62

^*a*^Molar mass of starting carbohydrate.

^*b*^Main peak range in ESI-MS following deuteration of carbohydrate [M + Na]^+^.

^*c*^Average deuteration as evaluated by ^1^H NMR.

ESI-MS revealed that each of the carbohydrates had an increase in mass from the single molecular ion to a heavier, distribution of peaks. It should be noted that the method used here only deuterates protons adjacent to a hydroxyl group, hence 100% deuteration of the carbohydrate is not possible (Scheme 1) and we obtained a mixture of deuterated products.^14^ For our intended application complete deuteration was, however, unnecessary with ease, scale of the synthesis and regio- and stereo-selectivity being the key requirements. ^1^H NMR confirmed deuteration by a clear decrease in the number of proton signals relative to non-exchanged peaks. The ^1^H NMR spectra of methyl-α-d-glucopyranoside are shown in Fig. 2 (Fig. S1–S5 for other sugars, ESI†) showing the change in peak intensity following deuteration to give ^2^H-methyl-α-d-glucopyranoside.

**Fig. 2:**
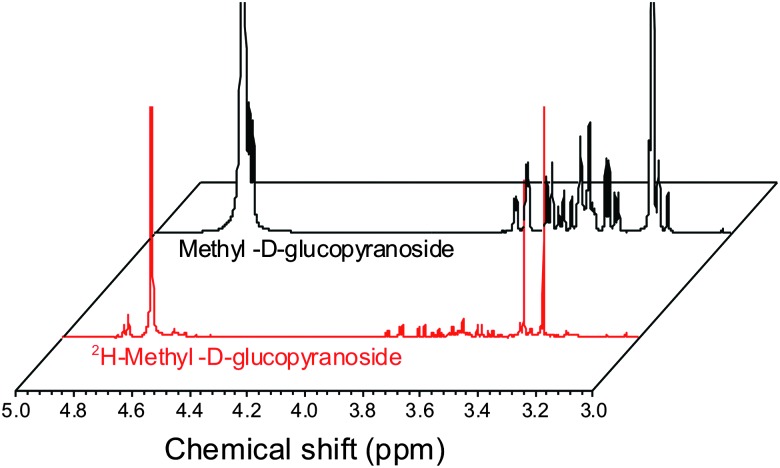
^1^H NMR spectra of methyl-α-d-glucopyranoside before and after deuteration, showing reduction in ring-proton intensity following deuteration, but retention of the methyl protons.

With this diverse range of ^2^H carbohydrates to hand it was possible to assess the uptake of these carbohydrates by *M. smegmatis* (a non-pathogenic model for *M. tuberculosis*) using NRA ion beam analysis. In an initial screening experiment, ^2^H trehalose was incubated with *M. smegmatis* at a final concentration of 50 mM for 60 minutes. After this time, the cells were pelleted, washed with PBS, heat-killed (95 °C, 15 min) and freeze-dried to provide a solid lyophilised sample suitable for subsequent ion beam analysis. The solid pellets were compressed to a homogenous disc and it was ensured that the thickness of this pellet was greater than the penetration depth of the ^3^He^+^ ion beam (∼4 microns). Full experimental details can be found in the ESI.† Briefly, the samples were irradiated with the ^3^He^+^ ion beam and protons resulting from the nuclear reaction were detected at 170°. The low natural abundance of ^2^H, along with the high energy of the detection of the emitted protons meant that this gave extremely good signal to noise ratio, and is ideal to detect low levels of deuterated carbohydrates. Encouragingly, the ion beam analysis detected the deuterium signal of the ^2^H labelled *M. smegmatis* heat-killed cells, compared to zero signal for cell-only *M. smegmatis* control. This indicated that the amount of ^2^H trehalose being taken up by *M. smegmatis* is in a range that is detectable by this method. Lysis of the cells and ion-beam analysis of the cytosolic fraction confirmed that ^2^H trehalose was taken up by *M. smegmatis* and not just bound/immobilised to the cell wall (data not shown).

To probe the utility of this new analytical method, the panel of sugars in Table 1 were investigated for uptake into *M. smegmatis*. The ability to probe such a panel of sugars is crucial to gain information about the carbon sources employed by mycobacteria that are transported into the cell and metabolised both *in vitro* and *in vivo.* The ^2^H carbohydrates were again added at a final concentration of 50 mM and incubated with *M. smegmatis* for 60 minutes. Following acquisition using NRA and analysis of the data we were able to calculate the relative molar uptake of each ^2^H-carbohydrate as shown in Fig. 3. This calculation included a correction for the total level of deuteration per-sugar (determined from Table 1) to ensure that a direct comparison of uptake of each ^2^H carbohydrate can be made.

**Fig. 3:**
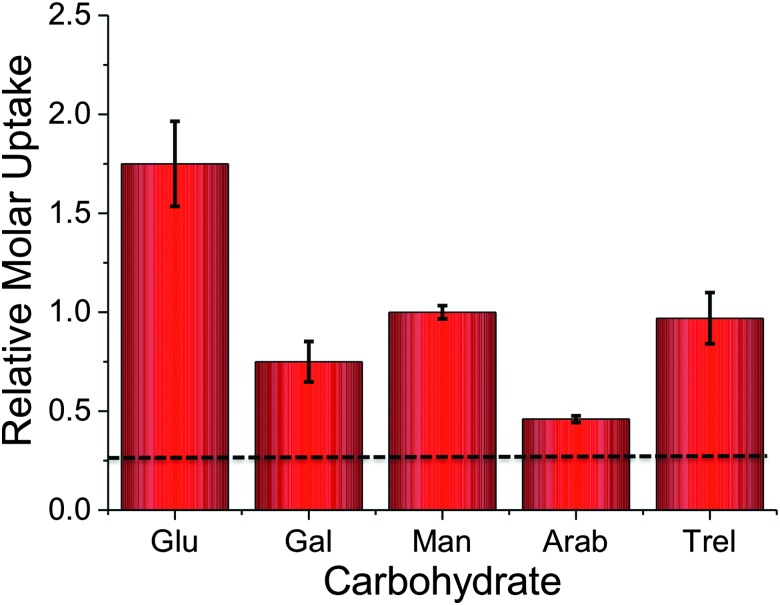
Relative molar uptake of carbohydrates into *M. smegmatis* determined by ion beam analysis. Uptake corrected for relative degrees of deuteration. Dashed line is the background signal from the cell-only fractions.

The results obtained from the NRA ^2^H-carbohydrate uptake assay are comparable to those utilising either ^14^C carbohydrates, fluorescently labelled carbohydrates or azido-modified carbohydrates.^2–4^ Additionally our results agree with previous studies where mycobacteria were grown on carbohydrates as the sole carbon source.^15,16^ It should be highlighted that the metabolic utilisation of carbohydrates by mycobacteria will differ from study to study depending on the experimental conditions used.

Recently, it has been shown that trehalose is recycled from the cell wall of *M. tuberculosis* and is taken up by an ABC-transporter that is essential for the virulence of this pathogen.^2^ *In vitro* studies have demonstrated that trehalose can serve as a sole carbon source for mycobacteria and that it is an essential precursor for cell wall metabolites.^17^ Intriguingly, trehalose uptake has been shown to be tolerant to a range of chemically altered trehalose analogues indicating its potential role as a new drug and biosensing target.^3^ Given the important role of trehalose in mycobacteria we sampled uptake of ^2^H trehalose by *M. smegmatis* from 0–60 min. A clear increase in the uptake of ^2^H trehalose over the 60 minute time interval was found, with a significant uptake of ^2^H trehalose at 30 min, Fig. 4.

**Fig. 4:**
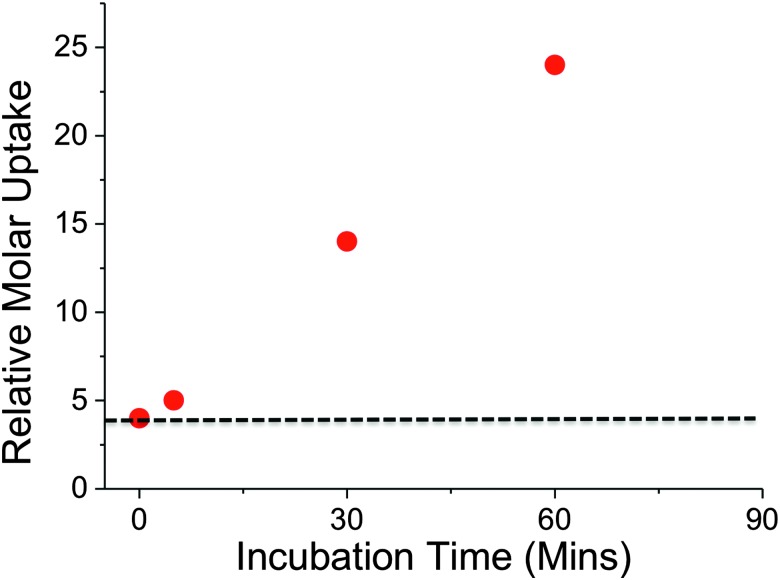
Time dependent uptake of ^2^H-trehalose into *M. smegmatis*. Dashed line indicates cell-only background.

Crucially, these results demonstrate that this NRA method can be used for monitoring dynamic uptake processes, especially with relatively slow growing organisms such as mycobacteria. A key feature to emphasise is the novel use of ion-beam analysis to evaluate biological uptake processes utilising deuterated probes that are structurally more analogous to the ‘native’ sugars, than chemically modified probes, such as FITC-modified-, azido-modified-sugars and are easier and cheaper to handle than radio-labelled carbohydrates.

In summary, we have taken advantage of the ability to deuterate carbohydrates in a facile, regio- and stereo-selective scalable manner enabling rapid access to a wide-range of carbohydrates that has allowed us to determine the relative uptake of a panel of ^2^H-carbohydrates by *M. smegmatis.* This ^2^H-carbohydrate uptake was probed experimentally and analysed by making use of the ^3^He nuclear reaction analysis using an ion-beam. To our knowledge this is the first use of such analysis in the discovery of small molecule uptake in bacteria and is comparable to data obtained by other methods. Using this method, the unusual uptake of trehalose into mycobacteria is observed, which is of particular importance in the development of new treatments and diagnostics for pathogenic mycobacteria such as *M. tuberculosis* and for probing carbohydrate uptake into a range of biotechnologically, and medically, relevant organisms.

We would like to thank Dr Adrian Chaplin for technical expertise. Equipment used was supported by the Innovative Uses for Advanced Materials in the Modern World (AM2), with support from Advantage West Midlands (AWM) and part funded by the European Regional Development Fund (ERDF). RL acknowledges the EPSRC MOAC doctoral training centre for a studentship. MIG was a Birmingham Science City Interdisciplinary Research Fellow funded by the Higher Education Funding Council for England (HEFCE). EF had a Leverhulme Trust Early Career Fellowship and now has a Sir Henry Dale Fellowship jointly funded by the Wellcome Trust and the Royal Society (Grant Number 104193/Z/14/Z).
