# Supplementary material for: Deuterated carbohydrate probes as ‘label-free’ substrates for probing nutrient uptake in mycobacteria by nuclear reaction analysis
Source: Chem Commun (Camb). 2015 Feb 19;51(23):4838–41. doi: 10.1039/c4cc09588j (PMC4774366; doi:10.1039/c4cc09588j)
Supplement: Supplementary file 1 [file CC-051-C4CC09588J-s001.pdf]

Electronic Supplementary Material (ESI) for ChemComm.
This journal is © The Royal Society of Chemistry 2015

Supporting Information for:

Deuterated Carbohydrate Probes as 'Label-Free' Substrates for Probing Nutrient Uptake in Mycobacteria by Ion Beam Analysis

R. Lowery,[a,b] M.I. Gibson,[b] R.L.Thompson[c] and E. Fullam*[a]

[a] School of Life Sciences, University of Warwick, Gibbet Hill Road, CV4 7AL, UK

[b] Department of Chemistry, University of Warwick, Gibbet Hill Road, CV4 7AL, UK

[c] Department of Chemistry, University of Durham, South Road, DH1 3LE, UK

Corresponding Author e-mail: e.fullam@warwick.ac.uk

Table of Contents

Experimental Section 2

General Methods and Materials 2

Physical and Analytical Methods 3

Typical Procedure for the Ru/C-Catalysed H-D Exchange of Sugars 4

Evaluation of deuterium content 5

Compound Data 6

Comparative ^{1}H NMR to demonstrate deuteration 9

ESI-MS of deuterated carbohydrates 14

Uptake experiments in *Mycobacterium smegmatis* 24

Nuclear Reaction Analysis 25

Supporting Information References 27

Experimental Section

General Methods and Materials

All chemicals were used as supplied unless otherwise stated. Ruthenium on carbon (5 wt. % loading), sucrose, TWEEN®80, phosphate buffered saline (PBS) tablets (1 tablet in 200 mL water to obtain 0.137 M NaCl, 2.7 mM KCl, 10 mM phosphate buffer solution, pH 7.4) and deuterium oxide (99.9 atom % D) were supplied by Sigma-Aldrich. α-α-D-Trehalose dihydrate, methyl-β-D-galactopyranoside, and methyl-α-D-mannopyranoside were supplied by Carbosynth. Methyl-β-L-arabinopyranoside was supplied by VWR International. Methyl-α-D-glucopyranoside was supplied by Fluka Analytical. Bacto Tryptic Soy Broth (TSB) was supplied by Scientific Laboratory Supplies Ltd. Methanol (laboratory reagent grade) was purchased from Fischer Scientific. 0.1mm glass beads were obtained from Thistle Scientific Ltd. High purity hydrogen gas (99.995 % minimum) was supplied by BOC gases.

Physical and Analytical Methods

NMR spectroscopy (^1H) measurements were conducted on either Bruker DPX-300 or DPX-400 spectrometers using deuterium oxide as a solvent. All chemical shifts are reported in ppm (δ) relative to the residual solvent. All spectra were acquired using a zg30 pulse sequence with a minimum of 16 scans. Mass spectral analyses were recorded on an Agilent 6130B single Quad spectrometer using electrospray ionisation (ESI) in positive mode on samples prepared in methanol. Lyophilisation was conducted on a Heto lyolab 300 freeze dryer. Optical density measurements were conducted at a wavelength of 600 nm on a Shimadzu UVmini-1240 UV visible spectrophotometer. The ion beam accelerator is a National Electrostatics Corporation (USA) 5SDH Pelletron accelerator, having a target station equipped with a liquid nitrogen cooling system, based at the University of Durham, UK.

Typical Procedure for the Ru/C-Catalysed H-D Exchange of Sugars

Trehalose (1.5 mmol, 0.51 g) was dissolved into 2 mL of D_2O to which Ru/C was added (10 mol % relative to the substrate). The solution was stirred in a butyl crimp sealed reaction vessel, and degassed with hydrogen, after which a balloon filled with hydrogen gas was fitted to the vessel, to maintain a pressure of ~ 1 bar. The vessel was subsequently heated to 80 °C for 72 hours. After this time, the mixture was cooled to room temperature and the solid catalyst removed using a syringe membrane filter (Sartorius Stedim, Minisart®, 0.2 μm). The filtrate was then lyophilised to yield a white solid.

This same procedure was followed for the deuteration of monosaccharides but with 5 mol % of 5 % Ru/C and heated at 80°C for 24 hours.

Evaluation of deuterium content

The incorporation of deuterium was demonstrated by using ESI mass spectrometry and the deuterium content was quantified by ^{1}H NMR by integration of the protons on the methyl protons of the methoxy group, relative to the residual, non-exchanged ring-protons. In the case of the disaccharide carbohydrate: trehalose, protons at the C1 (anomeric) position were utilised instead of the methyl protons.

Calculation for average deuteration of carbohydrate, Sawama *et al* [1])

$$\left(1 - \frac{Sum\ of\ integrals\ at\ deuterated\ proton\ enviroments}{Sum\ of\ total\ protons\ able\ to\ undergo\ deuteration}\right) * 100 = \%\ deuteration$$

For example; ^{2}H-Methyl-β-D-galactopyranoside

Proton positions $H^{2\text{-}4}$, H^6 are able to undergo deuteration (Compound data (2)) due to their proximity to the hydroxyl group on the adjacent carbon. Therefore the total protons of non-deuterated Methyl-β-D-galactopyranoside at these positions is equal to five.

$$\left(1 - \frac{H^2 + H^3 + H^4 + H^6}{5}\right) = 40.6\%\ deuteration$$

$$\left(1 - \frac{0.41 + 0.36 + 0.55 + 1.65}{5}\right) = 40.6\%\ deuteration$$

It should be noted here that this calculation provides an average of all the possible deuteration combinations and that for any given degree of deuteration per sugar (e.g. 2 per molecule) there exist many possible structures and this leads to increased complexity in the NMR spectra. This does not prevent the analysis since the overall % deuteration is required here for the NRA.

In order to convert the relative mols of carbohydrate taken up by *Mycobacterium smegmatis*, the NRA signal was divided by the average number of deuterium atoms per sugar to give the relative mol carbohydrate.

Compound Data

^{2}H-Methyl-α-D-glucopyranoside (1)

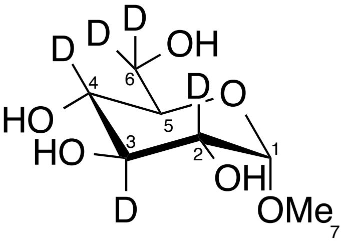

MS (ESI+) m/z: 218.0 $[M+Na+^2H]^+$, 219.0 $[M+Na+2x^2H]^+$, 220.0 $[M+Na+3x^2H]^+$

^{1}H NMR (400 MHz, D_2O) δppm: 3.85 (0.29H, dd, J=12.2, 2.3Hz, H^2), 3.82 (0.30H, dd, J=12.2, 5.4Hz, H^4), 3.75-3.69 (0.61H, m, H^6), 3.52 (0.30H, dd, J=9.7, 3.7Hz, H^4), 3.38 (1H, s, H^5), 3.31(3H, s, H^7)

^{2}H-Methyl-β-D-galactopyranoside (2)

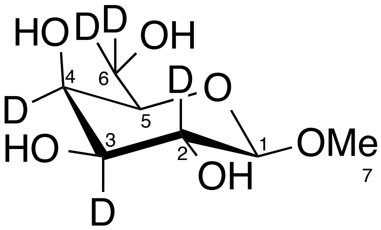

MS (ESI+) m/z: 218.0 $[M+Na+^2H]^+$, 219.0 $[M+Na+2x^2H]^+$

1H NMR (400 MHz, D_2O) δppm: 4.89 (0.27H, d, J=10.5Hz, H^1), 4.69 (0.73H, d, J=10.0Hz, H^1), 4.39 (0.41, d, J=7.8Hz, H^2) 4.00 (0.36H, dd, J=3.4Hz, H^3), 3.80 (1.66H, m, H^{4-6}), 3.65 (1H, s, H^5), 3.56 (0.57H, m ,H^{4-6}), 3.43 (3H, s, H^7)

^{2}H-Methyl-α-D-mannopyranoside (3)

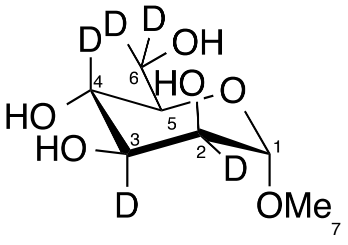

MS (ESI+) m/z: 218.0 $[M+Na+^2H]^+$, 219.0 $[M+Na+2x^2H]^+$

^{1}H NMR (400 MHz, D_2O) δppm: 4.71-4.87 (1H, m, H^1), 3.62-4.02 (2.51H, m, $H^{2\text{-}4,6}$), 3.48 (1H, s, H^5), 3.42 (3H, s, H^7)

^{2}H-Methyl-β-L-arabinopyranoside (4)

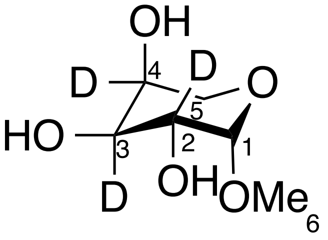

MS (ESI+) m/z: 188.0 $[M+Na+^2H]^+$, 189.0 $[M+Na+2x^2H]^+$, 190.0 $[M+Na+3x^2H]^+$

^{1}H NMR (400 MHz, D_2O) δppm: 4.90 (1H, d, J=2.9Hz, H^1), 4.07 (0.08H, s, H^3), 3.90-3.96 (2.46, m, $H^{4,5}$), 3.72 (1H, d, J=12.8Hz, H^3), 3.48 (3H, s, H^6)

^{2}H-Trehalose (5)

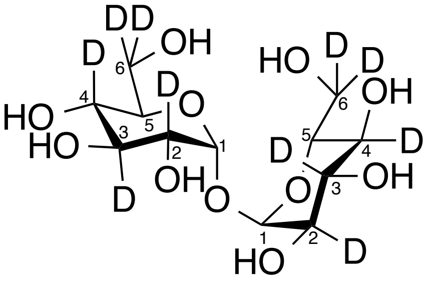

MS (ESI+) m/z: 366.0 $[M+Na+^{2}H]^{+}$, 367.0 $[M+Na+2x^{2}H]^{+}$, 368.0 $[M+Na+3x^{2}H]^{+}$, 369.0 $[M+Na+4x^{2}H]^{+}$

^{1}H NMR (400 MHz, D_2O) δppm: 5.14 (2H, s, H^{1}), 3.30-3.70 (7.42H, m, H^{2-6})

Comparative ^{1}H NMR to demonstrate deuteration:
(All spectra have been normalised relative to a non-deuterable peak.)

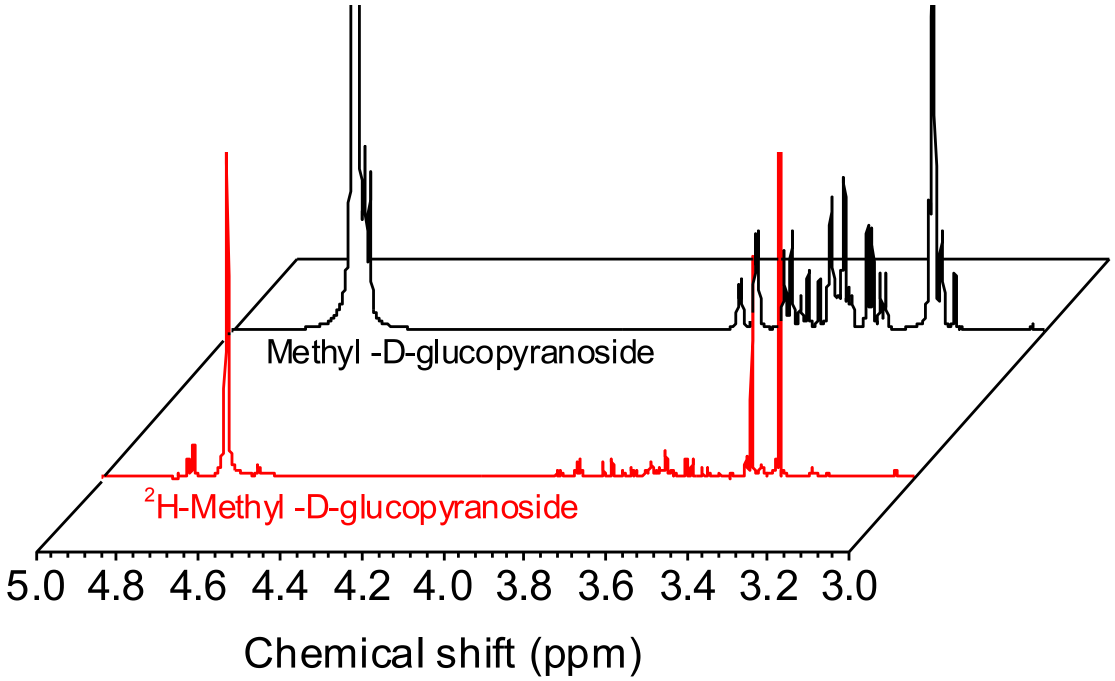

Figure S1 - ^{1}H-NMR spectrum comparing loss of signal intensity between ^{2}H-Methyl-α-D-glucopyranoside (red) and Methyl-α-D-glucopyranoside (black).

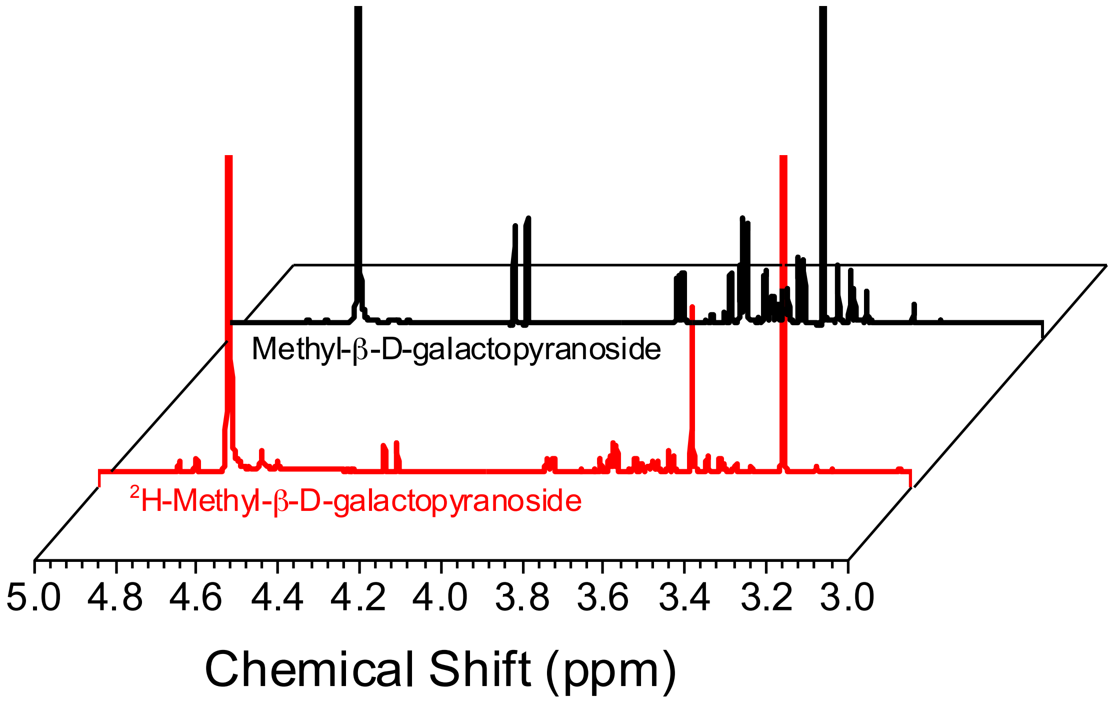

Figure S2 - ^{1}H-NMR spectrum comparing loss of signal intensity between ^{2}H-Methyl-β-D-galactopyranoside (red) and ^{2}H-Methyl-β-D-galactopyranoside (black).

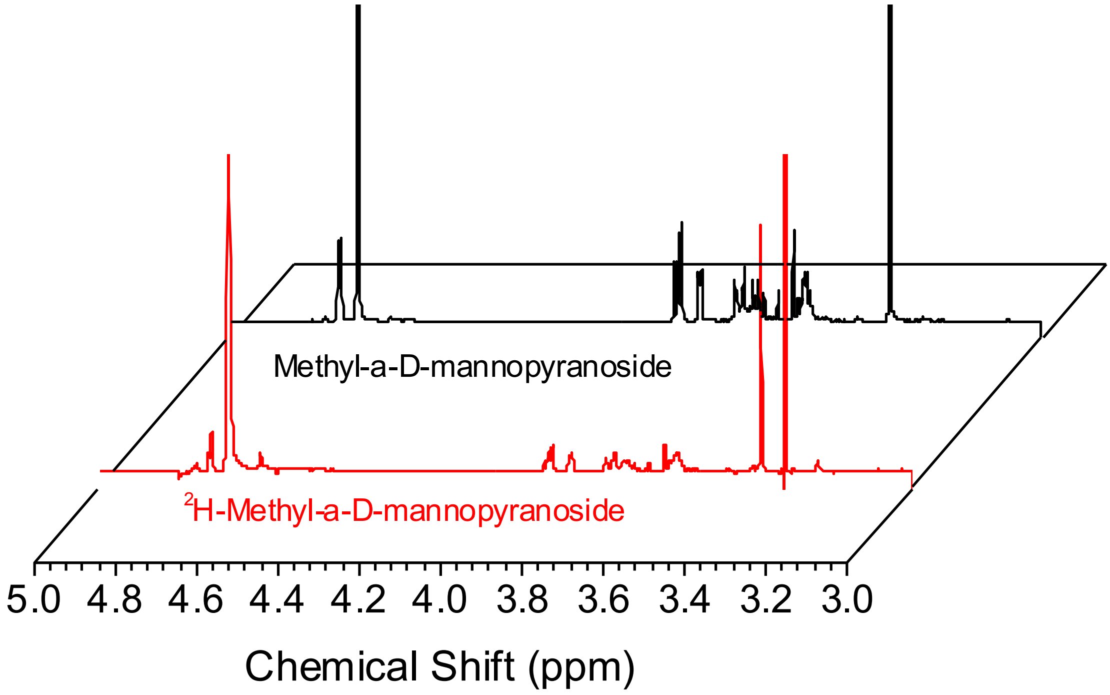

Figure S3 - ^{1}H-NMR spectrum comparing loss of signal intensity between ^{2}H-Methyl-α-D-mannopyranoside (red) and Methyl-α-D-mannopyranoside (black).

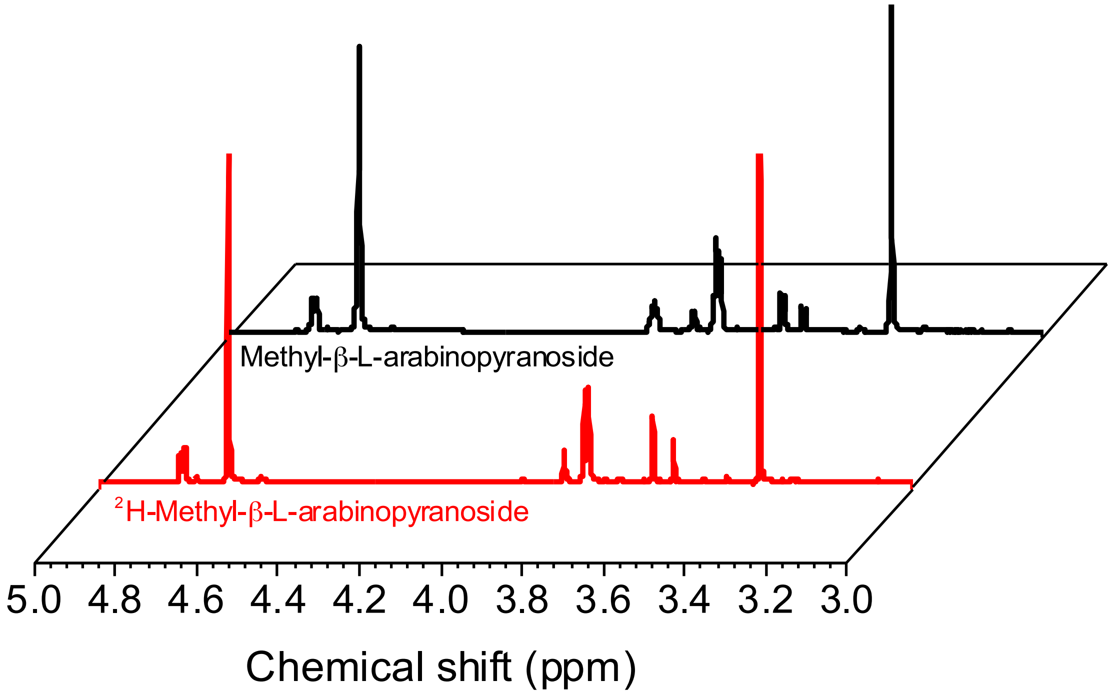

Figure S4 - ^{1}H-NMR spectrum comparing loss of signal intensity between ^{2}H-Methyl-β-L-arabinopyranoside (red) and Methyl-β-L-arabinopyranoside (black).

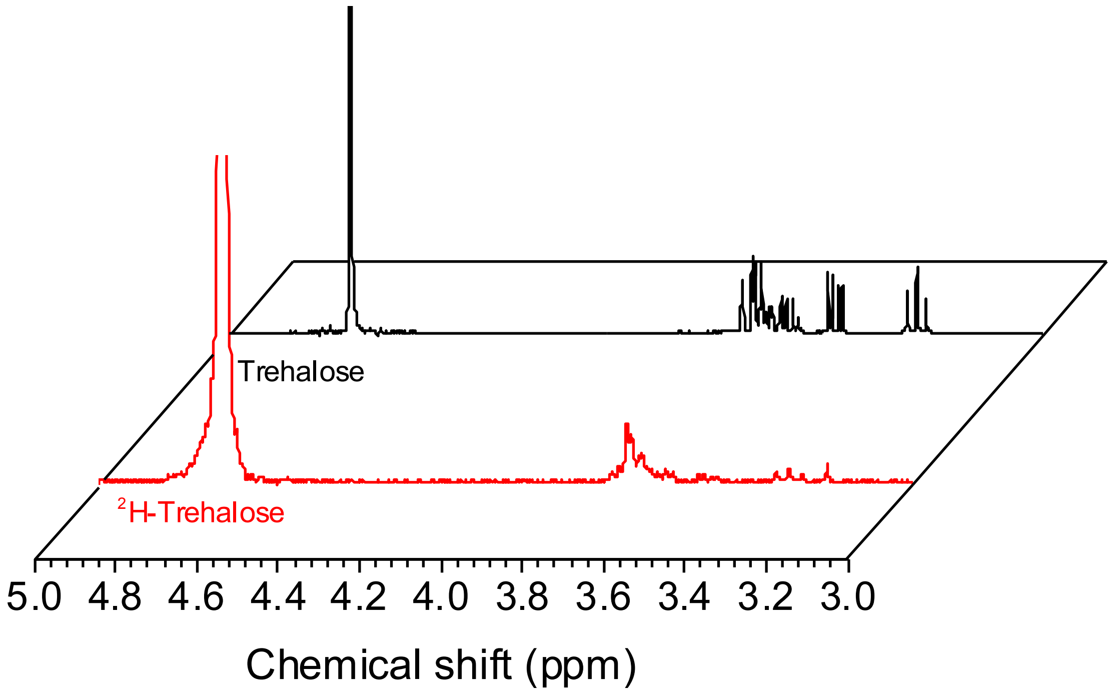

Figure S5 - ^{1}H-NMR spectrum comparing loss of signal intensity between ^{2}H-Trehalose (red) and Trehalose (black).

ESI-MS of deuterated carbohydrates

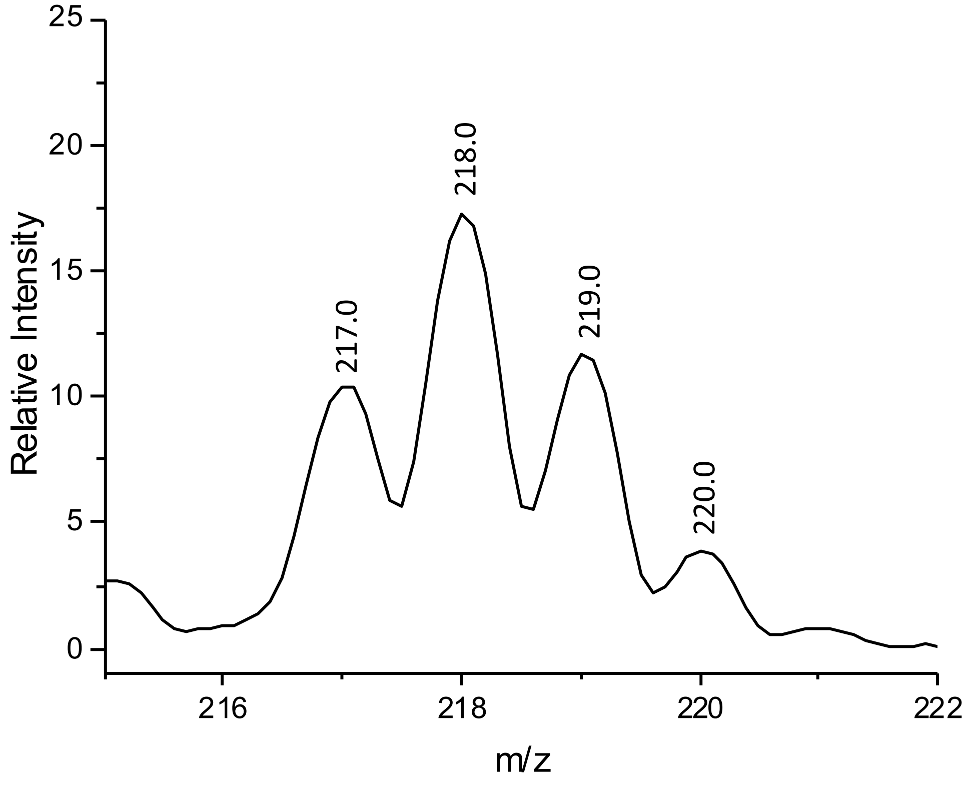

Figure S6 –ESI-MS (positive mode) spectrum of ^{2}H-Methyl-α-D-glucopyranoside

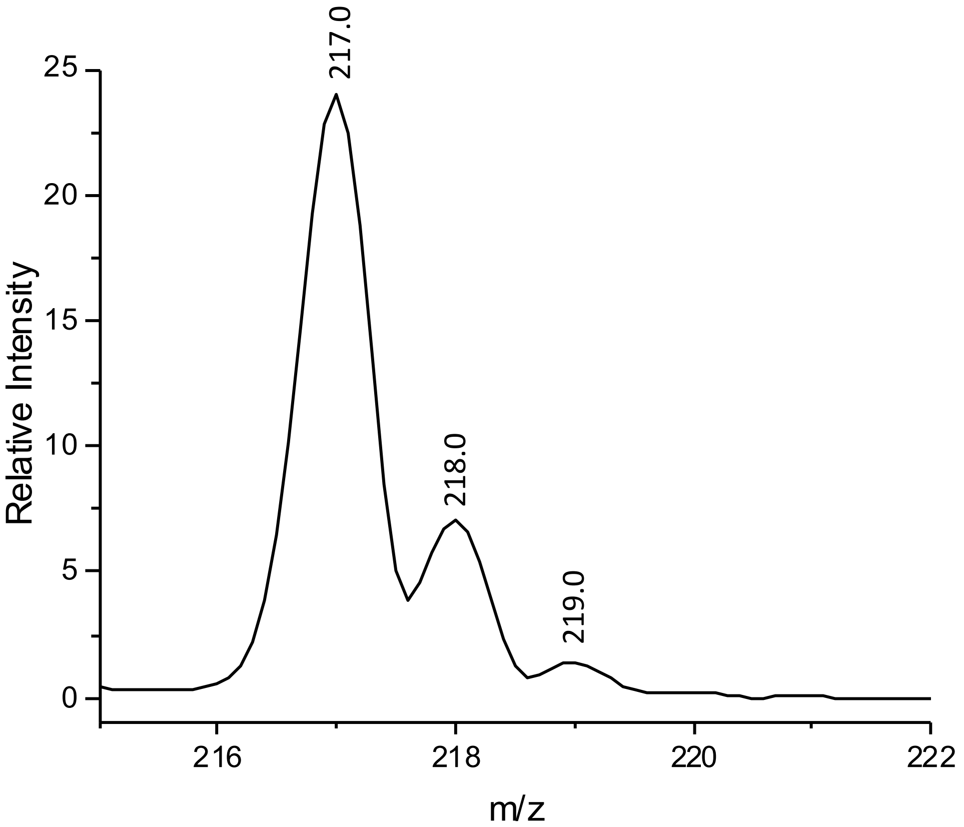

Figure S7 –ESI-MS (positive mode) spectrum of ^{2}H-Methyl-β-D-galactopyranoside

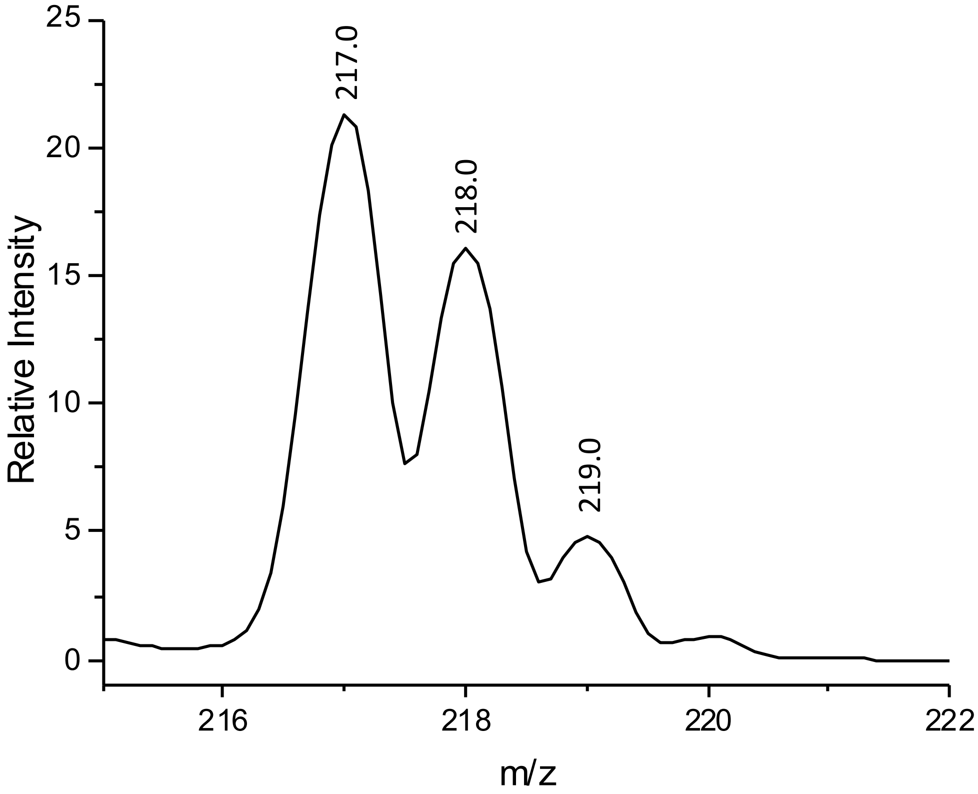

Figure S8 –ESI-MS (positive mode) spectrum of ^{2}H-Methyl-α-D-mannopyranoside

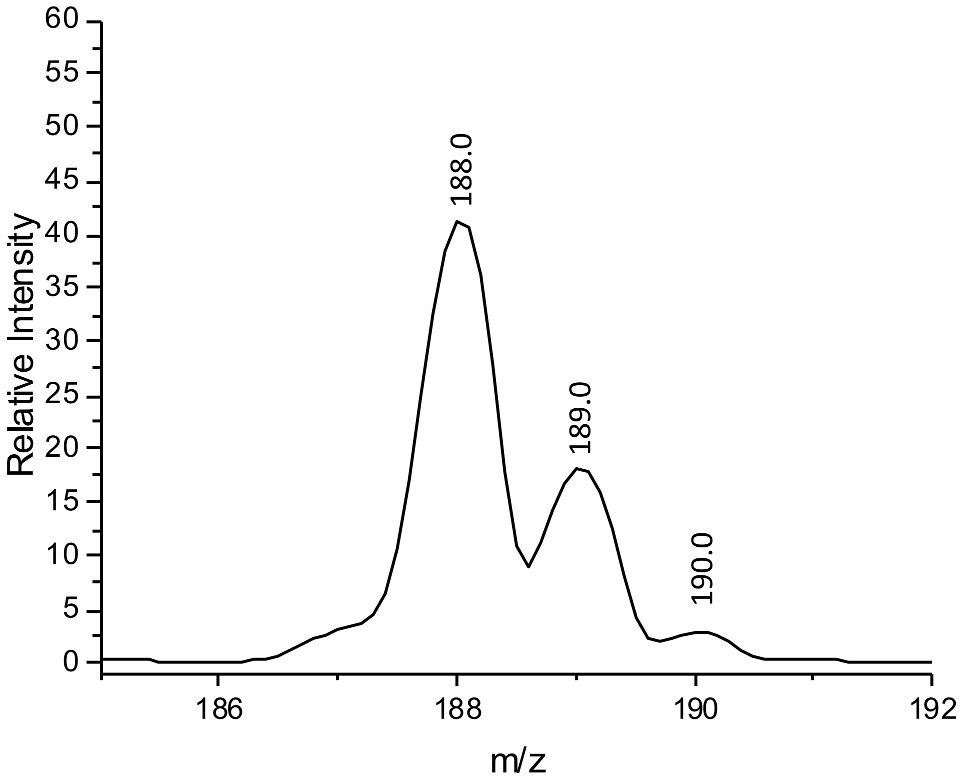

Figure S9 –ESI-MS (positive mode) spectrum of ^{2}H-Methyl-β-L-arabinopyranoside

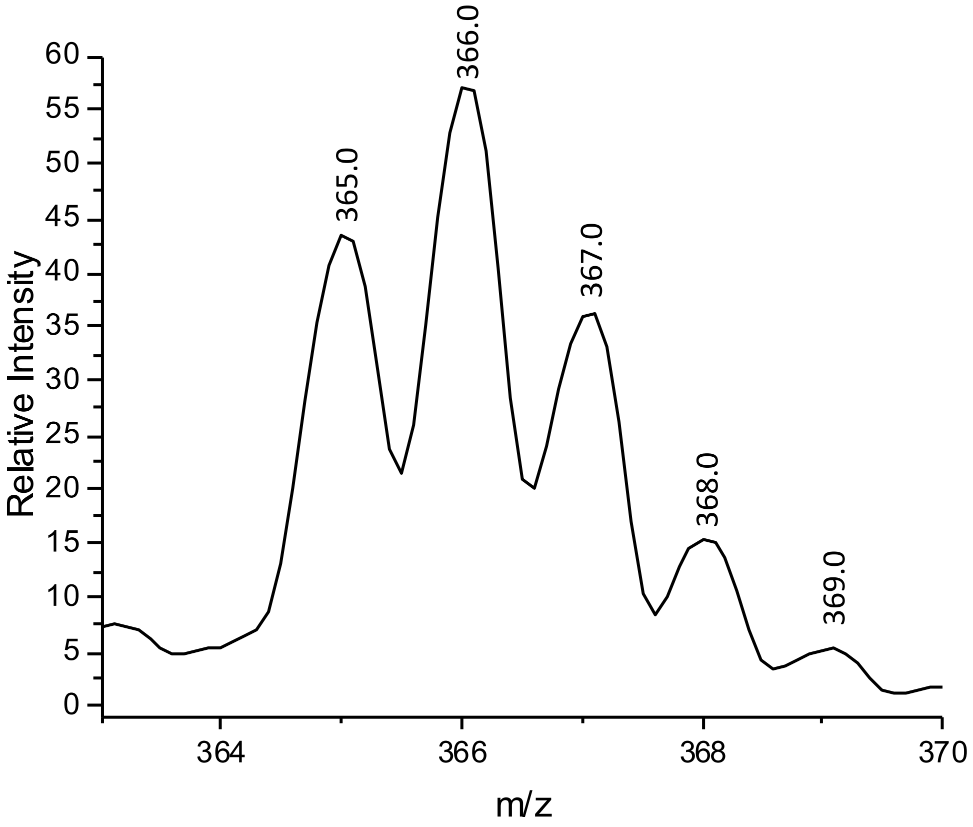

Figure S10 –ESI-MS (positive mode) spectrum of ^{2}H-Trehalose

Figure S11 - ^{1}H-NMR – 2H-Methyl-α-D-glucopyranoside

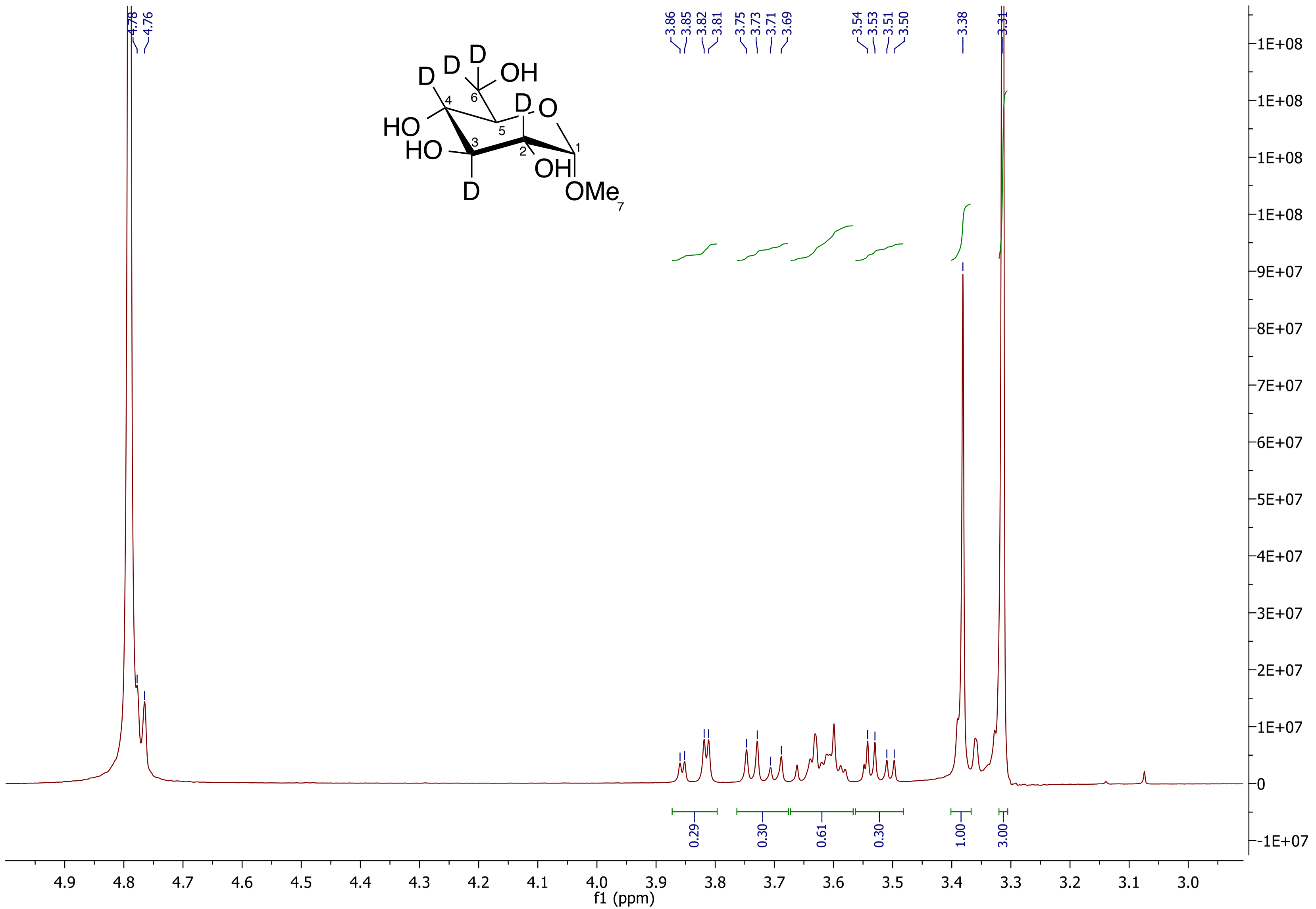

Figure S12 - ^{1}H-NMR – ^{2}H-Methyl-β-D-galactopyranoside

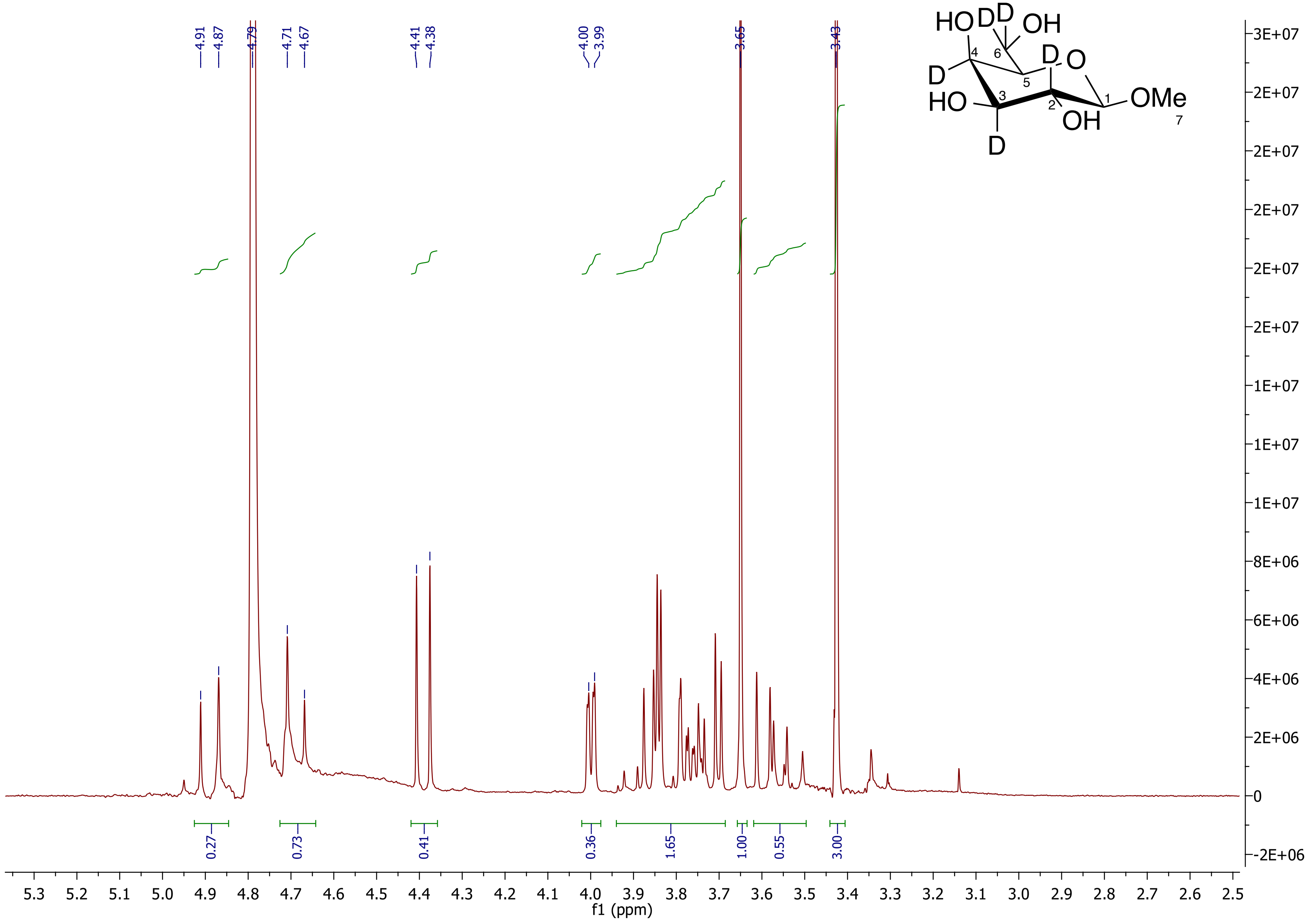

Figure S13 - ^{1}H-NMR – 2H-Methyl-α-D-mannopyranoside

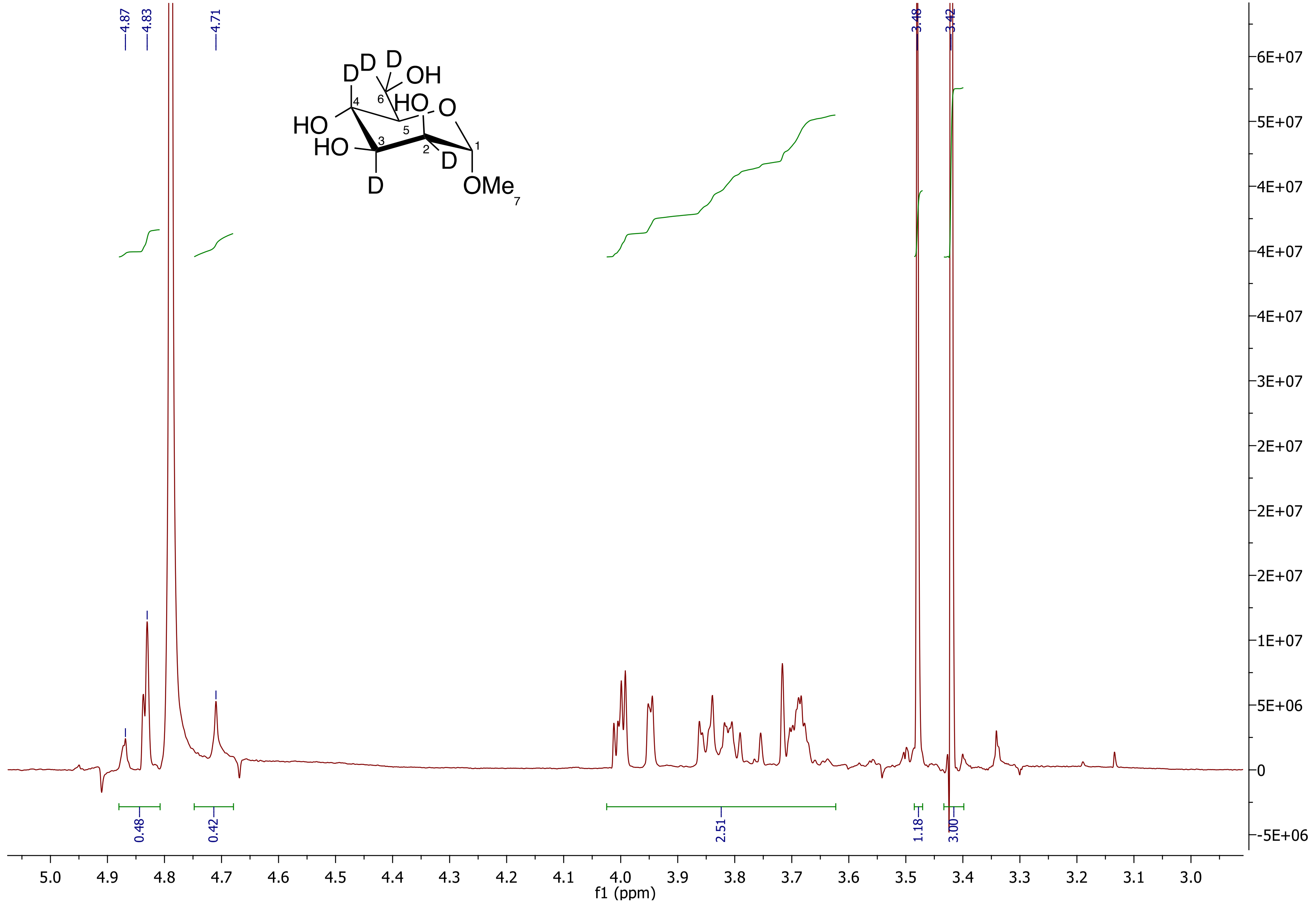

Figure S14 - ^{1}H-NMR – 2H-Methyl-β-L-arabinopyranoside

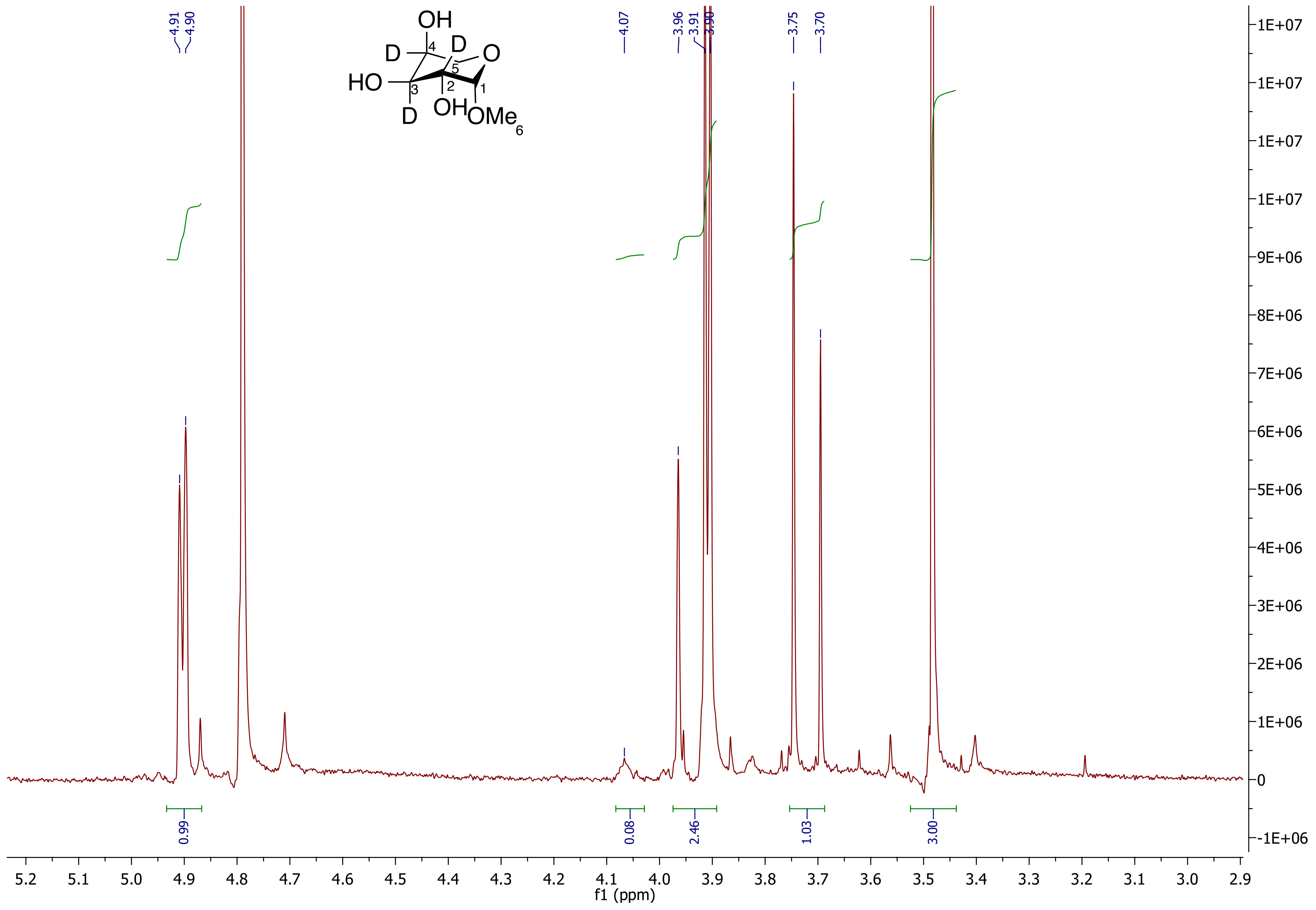

Figure S15 - ^{1}H-NMR – 2H-Trehalose

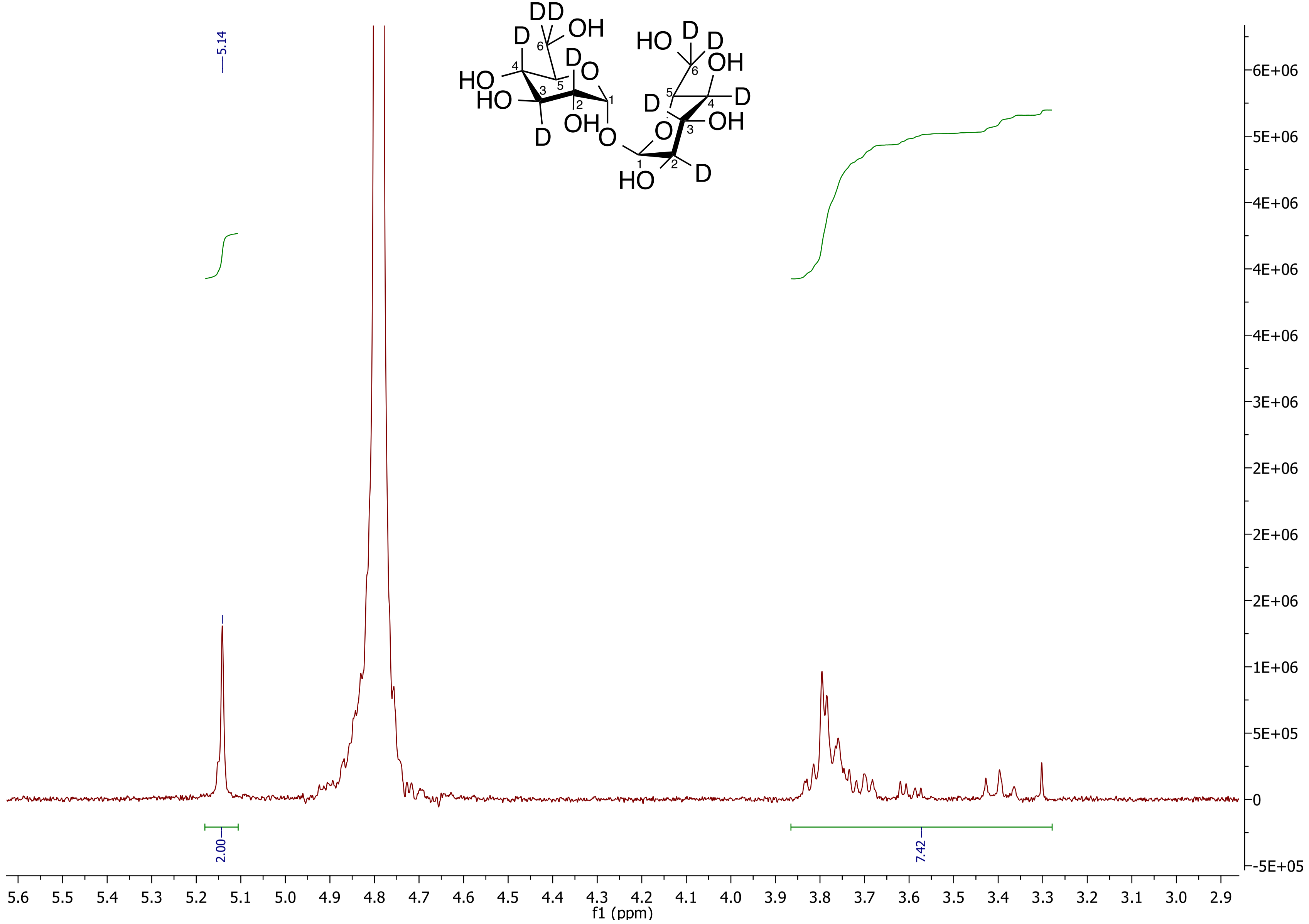

Uptake experiments in *Mycobacterium smegmatis*

An overnight culture of *Mycobacterium. smegmatis* mc^2155 was used to inoculate 1 L of Tryptic Soy Broth supplemented with 0.05% Tween 80. The 1 L culture was grown with shaking (180 rpm, 37°C) until the optical density at 600 nm (OD_{600}) had reached an OD_{600} of 0.9. The cells were then harvested by centrifugation (5000g, 20 mins, 4°C). The supernatant was removed and the cells from the 1 L culture were washed with PBS, centrifuged and resuspended in 20 mL PBS. The cells were aliquoted (950 µL) and used for each uptake experiment. The cells were incubated at 37°C, 800 rpm for 10 minutes before the addition of the deuterated sugars (**1-5**) at a final concentration of 50 mM. The cells were incubated at 37 °C, 800 rpm for 0-60 mins. At the required time point the cells were placed on ice and centrifuged (13,300 rpm, 2 mins). The cells were washed twice by re-suspending the pelleted cells in sterile ice cold PBS. The pelleted cells were subsequently resuspended in 100 µL of PBS and heat-killed at 95°C for 15 mins and subsequently lyophilised.

Nuclear Reaction Analysis

Lyophilised samples were pressed into pellets on aluminium foil using a press to prepare specimens that were homogenous and thick compared to the range (~4 microns) of the 0.7 MeV $^{3}He^{+}$ ion beam. The choice of beam energy coincident with a broad maximum in the nuclear reaction cross-section; therefore enables the most sensitive detection of deuterons. A defined charge of $^{3}He^{+}$ ions, typically 4 mC, was directed onto the sample at normal incidence and backscattered ions and reaction products were detected using a 1.5 mm thick Canberra PIPS detector with nominal resolution of 19 keV. Data analysis was carried out using the Surrey University Datafurnace programme, to provide a quantitative simulation of the fast proton spectrum under the instrument settings which we have established previously. Due to the relatively low count rate for the data obtained, and the homogenous nature of the samples, fitting to complex depth profiles was neither practical nor necessary. Instead the relative yield of fast protons was analysed to determine the relative content of ^{2}H-carbohydrate, since under the constant experimental conditions, these factors are directly proportional to one another. Example data and fitting is shown in Figure S16.

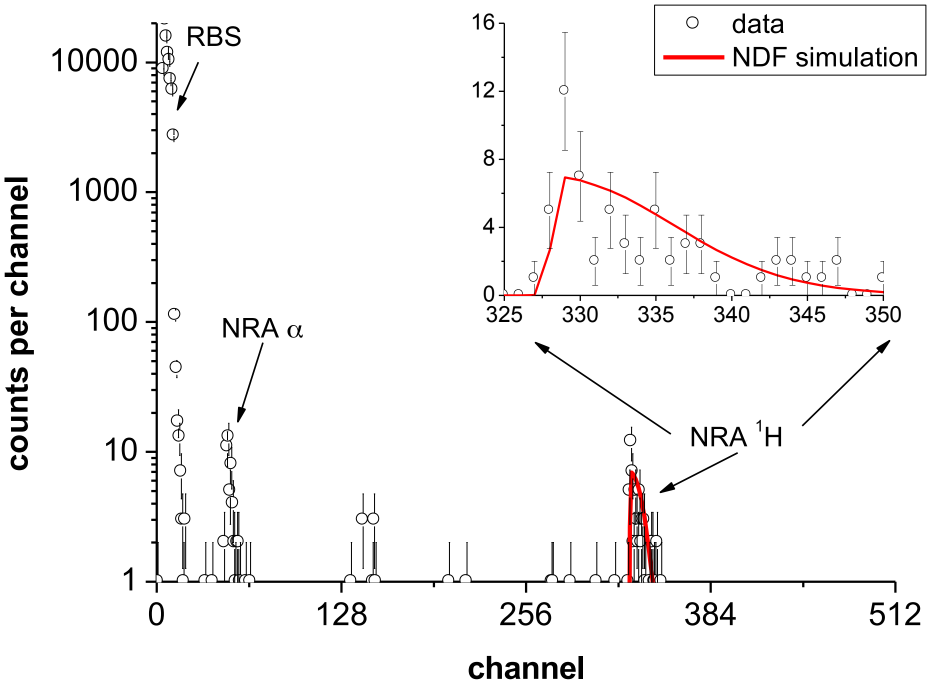

Figure S16 - $^{3}He^{+}$ ion beam analysis spectrum showing Rutherford backscattered (RBS) 3He and nuclear reaction products. The inset shows the proton nuclear reaction analysis (NRA) spectrum with simulation for 0.8% $^{2}H_{3.5}$-glucose in carbohydrate.

Supporting Information References

[1] Y. Sawama, Y. Yabe, H. Iwata, Y. Fujiwara, Y. Monguchi and H. Sajiki, *Chem. Eur. J.* **2012**, *18*, 16436-16442.
